# Excellent survival after R‐Hyper‐CVAD in hospitalized patients with high‐risk large B‐cell lymphoma: The Karolinska experience

**DOI:** 10.1002/jha2.296

**Published:** 2021-09-28

**Authors:** Kristina Sonnevi, Maria Ljungqvist, Jóel Kristinn Jóelsson, Sara Harrysson, Tove Wästerlid, Per Bernell, Björn Engelbrekt Wahlin

**Affiliations:** ^1^ Haematology Medical Unit Karolinska University Hospital Stockholm Sweden; ^2^ Division of Haematology, Department of Medicine, Huddinge Karolinska Institutet Stockholm Sweden; ^3^ Division of Haematology, Department of Medicine Karolinska Institutet Stockholm Sweden

**Keywords:** aggressive B‐cell lymphoma, chemotherapy, DLBCL, hyper‐CVAD, PMBCL, R‐CHOP, R‐Hyper‐CVAD

## Abstract

Patients with high‐risk aggressive B‐cell lymphoma exhibit poor survival after R‐CHOP. More intensive regimens yield higher rates of remission but also of complication. We investigated all 401 patients < 70 years with high‐risk (age‐adjusted [aa] international prognostic index [IPI] ≥2, extranodal, or bulky) aggressive B‐cell lymphoma hospitalized at Karolinska for urgent start of immunochemotherapy (129 R‐Hyper‐CVAD; 261 R‐CHOP/R‐CHOEP). Patients showed IPI 3–5 (70%), WHO PS ≥2 (49%), bulky disease (70%), extranodal (75%) and CNS (8%) involvement. Five‐year overall/progression‐free survival (OS/PFS) was better in patients who started R‐Hyper‐CVAD (84%/77%) compared with R‐CHOP/R‐CHOEP (66%/55%). Differences were independent in multivariable analysis, seen in all patient categories, and accentuated in extreme high‐risk disease: R‐Hyper‐CVAD vs. R‐CHOP/R‐CHOEP showed 5‐year PFS 69% vs.40% in aaIPI 3 and 88% vs. 38% in CNS involvement. For validation, survival was compared between the two Karolinska sites and calendar periods. Survival was superior 2006–2010 at the site that introduced R‐Hyper‐CVAD/R‐MA 2006, identical at both sites 2011–2017 after the other site adopted R‐Hyper‐CVAD/R‐MA 2011, and excellent 2018–2020 when R‐Hyper‐CVAD/R‐MA use increased to 75% of patients. Despite considerable toxicity, also patients aged 61–69 years showed better survival with R‐Hyper‐CVAD/R‐MA. This is the largest single‐centre series of patients treated with R‐Hyper‐CVAD/R‐MA, showing favourable outcome in high‐risk aggressive B‐cell lymphoma.

## 1 INTRODUCTION

Diffuse large B‐cell lymphoma (DLBCL) and other aggressive CD20+ B‐cell lymphomas are curable with immunochemotherapy. However, patients belonging to high‐risk categories are more difficult to cure and therefore show inferior 5‐year overall survival (OS) after standard rituximab, cyclophosphamide, doxorubicin, vincristine, prednisone (R‐CHOP) therapy: with international prognostic index (IPI) 4–5 54%, [1] with National Comprehensive Cancer Network IPI‐IPI 6–8 49%, [1] and in patients ≤60 years with age‐adjusted IPI (aaIPI) 3 40%. [2] Augmenting R‐CHOP with etoposide (E) appears to improve OS, [3, 4] but dose escalations of rituximab, cyclophosphamide, doxorubicin, vincristine, etoposide, prednisone (R‐CHOEP) have not been successful. [5] The addition of high‐dose IV methotrexate (HD‐Mtx) and high‐dose IV cytarabine (HD‐cytarabine) to R‐CHOP/R‐CHOEP correlates with better OS in retrospective analysis. [2]

This unmet clinical need in high‐risk patients has been explored in several prospective trials. In IPI 3–5, R‐DA‐EPOCH shows marginally better progression‐free survival (PFS), but not OS, than R‐CHOP. [6] Two Nordic Lymphoma Group (NLG) trials have shown promising results by adding HD‐Mtx and HD‐cytarabine courses to R‐CHOP and R‐CHOEP with 3‐year OS/PFS 81%/65%^7^ and 5‐year OS/PFS 83%/81%^8^; however, those trials excluded patients with central nervous system (CNS) involvement or world health organization (WHO) performance status (PS) 4. The French regimen using R‐ACVBP + autologous stem cell transplantation (ASCT) showed 4‐year OS/PFS 78%/76% in patients with aaIPI ≥2. [7] R‐CODOX‐M/R‐IVAC was recently tried in Britain with good results (3‐year PFS 75% for young patients) in IPI 3–5. [8] rituximab, hyperfractionated cyclophosphamide, vincristine, doxorubicin, dexamethasone (R‐Hyper‐CVAD)/rituximab, HD‐Mtx, HD‐cytarabine (R‐MA), originally developed for acute lymphocytic leukaemia (ALL), has also been tried for patients with WHO PS 0–2, with an excellent 3‐year PFS 87% in patients ≤45 years with aaIPI ≥2, but a high 12% treatment mortality in patients > 45. [9]

Thus, for patients with high‐risk disease, the optimal therapy has not been decided. We wanted to examine outcome (OS and PFS) and toxicity in high‐risk patients who started treatment hospitalized with R‐CHOP, R‐CHOEP and Karolinska's most intensive standard regimen, R‐Hyper‐CVAD.

## 2 METHODS

### 2.1 Patients

Using the Karolinska University Hospital records, we identified all patients aged < 70 years with aggressive CD20+ lymphoma who were hospitalized when receiving the first course of immunochemotherapy between 2002 and 2020. Hospitalized patients comprise the most urgent and high‐risk cases, with short diagnosis‐to‐treatment interval. [10] Patients had been admitted because of symptomatic disease with pressing need of intervention and/or high risk for tumour lysis syndrome. From electronic medical files we extracted information on clinical characteristics, treatment, toxicity and long‐term outcome. This retrospective, single‐centre, observational study excluded patients with Burkitt, primary CNS and transformed lymphoma, and those who did not show any objective sign of high‐risk disease (aaIPI ≥2, extranodal involvement, or bulk > 6 cm). The diagnostic biopsies were reviewed by Karolinska's haematopathologists per routine clinical practice. Cell‐of‐origin was determined using the Hans algorithm. [11] This study was approved by the Ethics Committee, Stockholm (2012/783‐31/3 with amendment 2016/2379‐32).

### 2.2 Aims

We aimed to evaluate the impact of first‐line therapy on PFS and OS in initially hospitalized patients with high‐risk aggressive B‐cell lymphoma, by investigating A. intention‐to‐treat analysis of immunochemotherapy and B. completed first‐line regimen. The first cycle of chemotherapy in these patients was either R‐CHOP, R‐CHOEP or R‐Hyper‐CVAD. Reflecting the real‐world nature of our material, some patients switched first‐line regimens after the first cycle, for various reasons, such as late‐coming information from the diagnostic biopsies or from liquor samples and toxicities (or absence thereof). We therefore conducted the secondary survival analysis on patients who had received full first‐line treatment. Since R‐Hyper‐CVAD/R‐MA use had varied over time and across Karolinska sites, we also explored survival differences in sites or calendar periods over R‐Hyper‐CVAD/R‐MA use, as a way to address patient‐selection bias. By doing this we aimed to estimate the impact of treatment intensity on survival also on group level to reduce the effect of selection bias in treatment decisions. The immunochemotherapy courses are described in supplement.

### 2.3 Choice of treatment and definitions of completed treatment regimens

R‐Hyper‐CVAD allocation was, originally, an individual decision made at one Karolinska site by the attending senior consultants, primarily in youngish patients with high‐risk markers such as CNS involvement, extensive extranodal disease, disease bulk, *MYC* translocation, double/triple hit, high‐grade B‐cell lymphoma (HGBCL) and primary mediastinal B‐cell lymphoma (PMBCL). Based on accumulated experience, the use of R‐Hyper‐CVAD later expanded to older patients, and clinical high‐risk scores were increasingly considered indications on their own for R‐Hyper‐CVAD. R‐Hyper‐CVAD/R‐MA was always planned for 6–8 courses, but patients who could not tolerate this were switched to R‐CHOP or R‐CHOEP to complete at least six cycles in first line. Other patients, having finished six cycles of R‐CHO(E)P, received consolidating cycles of HD‐Mtx and HD‐cytarabine (usually one each, based on an NLG trial Karolinska had participated in [12]). In the analysis of completed first‐line regimens, R‐CHO(E)P was defined as ≥6 courses of immunochemotherapy (at least 5 R‐CHO(E)P) and no HD‐MTX/cytarabine, R‐MA, or ASCT. R‐CHO(E)P + consolidation was defined as R‐CHO(E)P plus consolidating HD‐MTX/HD‐cytarabine, or ASCT (at least six courses). R‐Hyper‐CVAD/R‐MA was defined as ≥6 courses of immunochemotherapy, of which ≥2 R‐Hyper‐CVAD/R‐MA.

### 2.4 Therapy over time

The Karolinska University Hospital has two large academic tertiary sites, Solna and Huddinge, and Karolinska's Haematology Unit maintains one ward at either site. Between 2002 and 2017 aggressive lymphomas were managed at both wards; allocation was based on patients’ place of residence (north of the Royal Castle mid‐town: Solna, south: Huddinge). In 2006, Solna started treating high‐risk non‐Burkitt aggressive B‐cell lymphomas with R‐Hyper‐CVAD/R‐MA. Meanwhile, at Huddinge, the intensive approach for this category was 6 R‐CHOEP + 1 HD‐cytarabine + 1 HD‐Mtx. In 2011, Huddinge also began using R‐Hyper‐CVAD/R‐MA for high‐risk aggressive B‐cell lymphoma patients. At the end of 2017, the haematology unit undertook a centralization: from 2018 all lymphoid malignancies are treated at Solna.

### 2.5 Statistical analysis

Survival was calculated from date of diagnosis (defined as the date of obtaining diagnostic biopsy) until date of death (OS), death or progression of disease (PFS), except in the analysis of full regimens, where it was counted from the final day of the first‐line immunochemotherapy regimen. Last follow‐up was in April 2021. Univariate and multivariable analyses were conducted using Kaplan–Meier curves and Cox regression; the proportional hazards assumption was checked with graphs based on Schoenfeld residuals. Independent variables were assessed for correlations; depending on the nature of the variables, relationships between them were investigated using the Fisher's exact, Wilcoxon or Spearman tests. All *p* values are two‐tailed and calculated using Stata 14.2 (StataCorp, College Station, TX, USA). The forest plot was made with Review Manager 5.4 (https://www.cochrane.org). *p* < 0.05 was considered significant.

## 3 RESULTS

Between 2002 and 2020, the hospital records showed 401 patients < 70 years (median [range], 56 [17–69]) hospitalized for initiating urgent immunochemotherapy of a new CD20+ high‐risk aggressive B‐cell lymphoma. The patients were ill: 95% had symptomatic disease, 86% elevated lactate dehydrogenase, 84% stage III‐IV, 49% WHO PS 2–4, 70% IPI 3–5, 70% bulky disease, 75% extranodal involvement and 50% hypercalcaemia (Table 1). The median diagnosis‐to‐treatment interval was 8 days (p25, 4; p75, 15)—date of treatment defined as the first day of immunochemotherapy. After a median follow‐up of 7.5 years (range, 0.4–17.2), 136 patients had progressed, and 142 had died. The 5‐year OS/PFS was 70%/61%. CNS involvement at diagnosis was seen in 8% but, interestingly, not associated with inferior survival, while CD5 positivity, detected in 6%, was very adverse (Table 1). *MYC* translocations and double/triple hits were detected in 37 and 10 patients, respectively, but genetic investigations had only been conducted in 111 patients at the request of the attending clinician, so their true frequencies or impact could not be assessed; patients with double/triple hit showed 5‐year OS/PFS 64%/57% and those with only *MYC* translocation 72%/62%. The most common diagnoses were DLBCL (*n* = 285) and PMBCL (*n* = 40), followed by HGBCL (n = 35), post‐transplant lymphoproliferative disorder (*n* = 12), T cell/histiocyte‐rich large B‐cell lymphoma (*n* = 10), unspecified aggressive B‐cell lymphoma (*n* = 10), grey zone between Hodgkin and DLBCL (*n* = 4), follicular lymphoma grade 3B (*n* = 3) and lymphomatoid granulomatosis grade 3 (*n* = 2). Of the non‐intervention factors, unemployment/early retirement, Charlson comorbidity index, IPI, albumin and CD5 positivity were independent with respect to both OS and PFS; these variables competed with treatment regimens in the final multivariable Cox models.

**TABLE 1:** Patient characteristics at diagnosis

Factor			Correlation with outcome			
			Overall survival		Progression‐free survival	
	*N*	per cent	HR	*p* value	HR	*p* value
Age > 60 years	154	38%	2.7	<0.00005	1.9	<0.00005
Male sex	235	59%	1.3	0.18	1.3	0.096
No cohabitation with other adult (loneliness)	116	29%	1.3	0.19	1.5	0.011
Unemployed or early retired	54	13%	3.5	<0.00005	2.4	<0.00005
Smoking	86	21%	1.5	0.043	1.2	0.32
Substance abuse	18	4%	3.5	<0.00005	2.5	0.001
Severe psychiatric disorder	17	4%	2.4	0.003	2.7	0.0004
Charlton comorbidity index ≥ 2	52	13%	2.6	<0.00005	2.4	<0.00005
Human immunodeficiency virus (HIV) positivity	8	2%	1.3	0.66	1.3	0.58
WHO performance status 2–4	196	49%	2.4	<0.00005	2.2	<0.00005
B symptoms	217	55%	1.4	0.042	1.5	0.014
Other symptoms	371	93%	2.2	0.056	3.0	0.008
Haemoglobin < 100 g/l	98	24%	1.6	0.014	1.6	0.004
Platelets < 100/nl	30	7%	3.4	<0.00005	2.3	0.0003
Creatinine elevated	90	23%	2.1	<0.00005	1.9	0.0001
Lactate dehydrogenase (LDH) Elevated	345	86%	1.6	0.11	1.5	0.079
Missing	6	2%	1.8	0.32	1.5	0.53
Albumin <35 g/l	261	65%	2.4	<0.00005	2.3	<0.00005
Missing	10	2%	6.0	0.0001	4.1	0.0003
Calcium ≥2.60 mmol/l (albumin‐corrected)	199	50%	1.8	0.001	2.0	<0.00005
Missing	19	5%	2.8	0.002	2.4	0.003
Ann Arbor Stage III‐IV	338	84%	2.4	0.003	2.3	0.001
Disease bulk > 6 cm	278	70%	1.0	0.85	1.3	0.19
Number of extranodal sites						
0	102	25%	1		1	
1	132	33%	1.5	0.060	1.3	0.23
2–6	167	42%	1.5	0.073	1.4	0.077
CNS involvement	30	8%	1.2	0.65	0.8	0.45
Testis/ovarium involvement	15	4%	2.1	0.043	2.1	0.024
Bone marrow involvement	76	19%	2.2	<0.00005	1.9	0.0001
IPI						
0–1	44	11%	1		1	
2	75	19%	1.6	0.27	1.5	0.28
3–5	281	70%	3.6	0.001	3.7	0.0001
Missing	1	0%	NA		NA	
Age‐adjusted IPI						
0–1	60	15%	1		1	
2	181	45%	1.3	0.40	1.7	0.059
3	154	38%	3.4	<0.00005	3.7	<0.00005
Missing	6	2%	2.4	0.18	2.2	0.22
CNS‐IPI						
0–1	44	11%	1		1	
2–3	203	51%	2.2	0.050	2.5	0.009
4–6	150	37%	5.0	0.0001	4.3	<0.00005
Missing	4	1%	1.5	0.71	1.2	0.91
Diagnosis						
Diffuse large B‐cell lymphoma	285	71%	1		1	
Primary mediastinal B‐cell lymphoma	40	10%	0.2	0.002	0.4	0.006
Other subtypes	76	19%	0.7	0.069	0.8	0.26
Non‐GC according to Hans' algorithm	84	33%	1.1	0.53	1.3	0.23
CD5 positivity	23	6%	3.4	<0.00005	2.5	0.001

Abbreviations: aa, age‐adjusted; DLBCL, diffuse large B‐cell lymphoma; GC, germinal centre; HR, hazard ratio; IPI, international prognostic index; PTLD, post‐transplant lymphoproliferative disorder.

### 3.1 Intention‐to‐treat analysis

**FIGURE 1:**
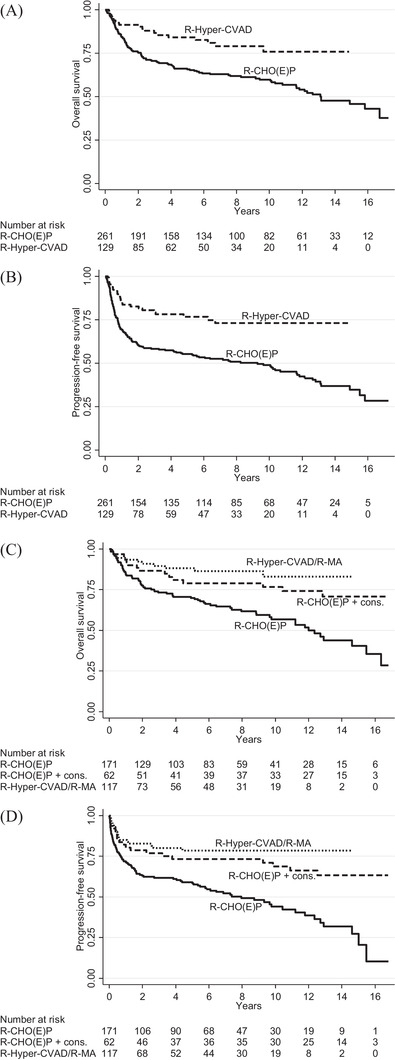
Overall survival (A) and progression‐free survival (B) by first course of immunochemotherapy. Overall survival (C) and progression‐free survival (D) by full first‐line regimen of immunochemotherapy

After admission, the first course of immunochemotherapy was 201 (50%) R‐CHOP, 60 (15%) R‐CHOEP (including 6 R‐DA‐EPOCH), 129 (32%) R‐Hyper‐CVAD and 11 (3%) other intensive R‐ALL courses which included rituximab (Table 2). R‐CHOP was used as the reference. A first course of R‐Hyper‐CVAD or R‐CHOEP instead of R‐CHOP correlated with better outcome in univariate analysis; in multivariable analysis, R‐Hyper‐CVAD, but not R‐CHOEP, remained an independent factor for better outcome (Table 2). R‐CHOP and R‐CHOEP were grouped as R‐CHO(E)P in subsequent analyses, and the few R‐ALL regimens not further investigated. The 5‐year OS/PFS was 84%/77% with R‐Hyper‐CVAD and 66%/55% with R‐CHO(E)P (Figure 1A,B).

**TABLE 2:** Immunochemotherapy and survival

		Correlation with overall survival						Correlation with progression‐free survival					
		Univariate analysis			Multivariable analysis			Univariate analysis			Multivariable analysis		
Analysis	*N*	HR	95% CI	*p* value	HR	95% CI	*p* value	HR	95% CI	*p* value	HR	95% CI	*p* value
**First course of immunochemotherapy**													
R‐CHOP	201	1			1			1			1		
R‐CHOEP	60	0.52	0.31–0.86	0.017	0.79	0.46–1.36	0.40	0.62	0.40–0.96	0.017	0.85	0.54–1.33	0.47
R‐Hyper‐CVAD	129	0.39	0.25–0.63	0.0002	0.58	0.35–0.94	0.030	0.37	0.25–0.56	0.0002	0.47	0.31–0.72	0.001
R‐ALL regimen	11	1.27	0.59–2.74	0.73	1.45	0.66–3.16	0.36	0.97	0.45–2.08	0.73	0.99	0.46–2.16	0.98
**Completed first‐line immunochemotherapy**													
R‐CHO(E)P	171	1			1			1			1		
R‐CHO(E)P + consolidation	62	0.44	0.25–0.77	0.004	0.49	0.27–0.89	0.019	0.43	0.26–0.70	0.001	0.47	0.28–0.79	0.004
R‐Hyper‐CVAD/R‐MA	117	0.33	0.18–0.59	0.0002	0.41	0.22–0.75	0.004	0.35	0.22–0.56	<0.00005	0.39	0.24–0.63	0.0001

Multivariable models were adjusted for unemployment/early retirement, Charlson comorbidity index, international prognostic index, albumin and CD5 positivity.

Abbreviation: HR, hazard ratio.

### 3.2 Differences between categories of therapy and clinical characteristics

**FIGURE 2:**
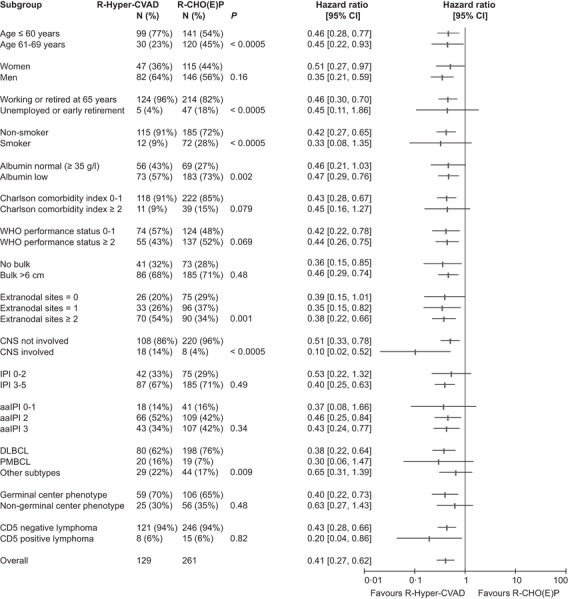
Distributions of clinical categories in patients who started with R‐Hyper‐CVAD and R‐CHO(E)P, and hazard ratio and 95% confidence interval (CI) in each category for each comparison of R‐Hyper‐CVAD and R‐CHO(E)P with respect to progression‐free survival, also shown in a forest plot Abbreviations: aa, age‐adjusted; DLBCL, diffuse large B‐cell lymphoma; IPI, international prognostic index; PMBCL, primary mediastinal B‐cell lymphoma.

Patients who started with R‐Hyper‐CVAD were younger than those starting with R‐CHO(E)P (median 51 vs. 60 years) but also showed more extensive extranodal involvement (54% vs. 34% had two or more sites), resulting in an equal distribution of high‐risk prognostic scores (Figure 2). This figure shows that R‐Hyper‐CVAD appeared superior to R‐CHO(E)P in all patient categories, also in those 61–69 years old. Absolute survival differences were more pronounced in extreme high‐risk disease. For example, R‐Hyper‐CVAD vs. R‐CHO(E)P showed 5‐year PFS 69% vs. 40% in aaIPI 3, 72% vs. 42% in IPI 4–5, 88% vs. 38% in CNS involvement and 66% vs. 7% in CD5+ lymphoma. The corresponding figures were 88% vs. 77% in aaIPI 0–1, 79% vs. 63% in aaIPI 2, 92% vs. 80% in IPI 0–1, 80% vs. 75% in IPI 2, and 74% vs. 52% in IPI 3. CNS involvement had at Karolinska been recognized as a strong indication for R‐Hyper‐CVAD, as had *MYC* translocation (67% of patients with *MYC* translocation received R‐Hyper‐CVAD), which explains why these factors were not associated with outcome overall, in contrast to CD5 positivity and bone‐marrow involvement, because the latter had not influenced therapeutic choice. Established IPIs retained some adverse prognostic impact in R‐Hyper‐CVAD starters, although the overall improved outcome reduced absolute differences, as shown above; however, extranodal involvement, including the CNS (see above), lost all negative bearing on PFS (*p* = 0.51). Survival estimates in DLBCL, PMBCL and other subtypes all favoured R‐Hyper‐CVAD (supplement), with 5‐year OS/PFS 86%/78% vs. 62%/53% (DLBCL) and 95%/89% vs. 89%/68% (PMBCL).

### 3.3 Completed first‐line regimen

Nineteen patients received only the first R‐Hyper‐CVAD course and then continued with R‐CHO(E)P; thus, they never received R‐MA. Conversely, 14 patients received 1–2 cycles of R‐CHO(E)P and then continued with R‐Hyper‐CVAD/R‐MA. Regimen switches occurred throughout the first‐line treatments. To investigate completed first‐line therapy, patients were categorized into three treatment groups: R‐Hyper‐CVAD/R‐MA (*n* = 117), R‐CHO(E)P + consolidation (*n* = 62) and R‐CHO(E)P (*n* = 171; Table 2). Of the full treatments, R‐Hyper‐CVAD/R‐MA correlated with superior survival, both in univariate and multivariate analysis (Figure 1; Table 2) with 5‐year OS/PFS 88%/78%. To a lesser extent, also R‐CHO(E)P + consolidation was associated with better outcome with 5‐year OS/PFS 79%/73%. Full R‐CHO(E)P showed 5‐year OS/PFS 71%/59% (Figure 1C,D). Five patients who for first‐line received highly individualized ALL‐inspired therapy were not further investigated. Some patients had < 6 cycles and thus did not receive a full first‐line chemotherapy regimen: 34/261 R‐CHO(E)P starters (13%) of whom 18 (7%) had primary refractory disease, 10 of 129 R‐Hyper‐CVAD starters (8%) of whom three (2%) had primary refractory disease and two of 11 R‐ALL regimen starters. These 46 patients showed, as expected, poor outcome with 5‐year OS/PFS 22%/16%.

### 3.4 The number of R‐Hyper‐CVAD/R‐MA cycles and toxicity

Out of 117 patients treated with complete R‐Hyper‐CVAD/R‐MA in first line, 87 received 6–8 cycles of R‐Hyper‐CVAD/R‐MA, 30 received two‐five cycles of R‐Hyper‐CVAD/R‐MA preceded or succeeded by R‐CHO(E)P to add up to a total of ≥6 cycles. The 5‐year OS/PFS was in those given six‐eight courses of R‐Hyper‐CVAD/R‐MA 89%/81%, in those given two‐five courses of R‐Hyper‐CVAD/R‐MA + R‐CHO(E)P 84%/72% (similar to R‐CHO(E)P + consolidation with 5‐year OS/PFS 79%/73%). In total, 150 patients received ≥1 cycle of R‐Hyper‐CVAD/R‐MA at some point during induction, with 5‐year OS/PFS 81%/74%. Patients starting R‐Hyper‐CVAD became older over time: median age was 41, 52 and 54 years in the three calendar periods (*p* = 0.015). Despite this, life‐threatening complication rates did not increase (22%, 19%, 13%), and a larger fraction of fully treated patients received six‐eight instead of two‐five courses of R‐Hyper‐CVAD/R‐MA over time (74%, 68%, 83%). Of the 30 elderly R‐Hyper‐CVAD starters, 13 received six‐eight cycles R‐Hyper‐CVAD/R‐MA, 11 two‐five cycles, six only one cycle. Toxicity was examined in the 150 patients who received at least one cycle of R‐Hyper‐CVAD/R‐MA: 11 (7%) showed mild, 82 (55%) severe and 26 (18%) life‐threatening complications. These most severe or life‐threating complications were in 90 patients infections (including three COVID‐19 cases), in five gastrointestinal toxicities (including two bowel perforations), in four neurologic toxicities, in four haemorrhages, in two prolonged cytopaenias, in two fatigue, in one tumour lysis syndrome. Six patients died from complications (4%; five sepsis, one haemorrhage), their median age was 60 (range, 54–65) years. Two died after the second cycle (R‐MA), one patient died after his second R‐CHOP, having switched from a first R‐Hyper‐CVAD cycle; three patients died after having switched to R‐Hyper‐CVAD/R‐MA from R‐CHOP. Among the 243 other patients who received R‐CHO(E)P (excluding eight patients who started with R‐ALL regimens), there were 19 (8%) mild, 81 (34%) severe and 27 (11%) life‐threatening complications; six of 243 (2%) died from toxicity (all from sepsis, median age 63).

Among 150 patients who had received ≥1 cycle of R‐Hyper‐CVAD/R‐MA, one developed a subsequent acute myeloid leukaemia. In the other 243 patients, there were three cases of secondary myeloid malignancy (the three had received five‐seven cycles of R‐CHOEP, and one had undergone ASCT), one each of acute myeloid leukaemia, myelodysplastic syndrome and chronic myelogenous leukaemia. The development of heart disease among patients treated with full first‐line was seen in six (5%) after R‐Hyper‐CVAD/R‐MA and in 28 (16%) after R‐CHO(E)P with or without consolidation; in patients ≤60 years, these figures were two (2%) and nine (7%).

### 3.5 Geographic and temporal clustering of R‐Hyper‐CVAD/R‐MA patients

**FIGURE 3:**
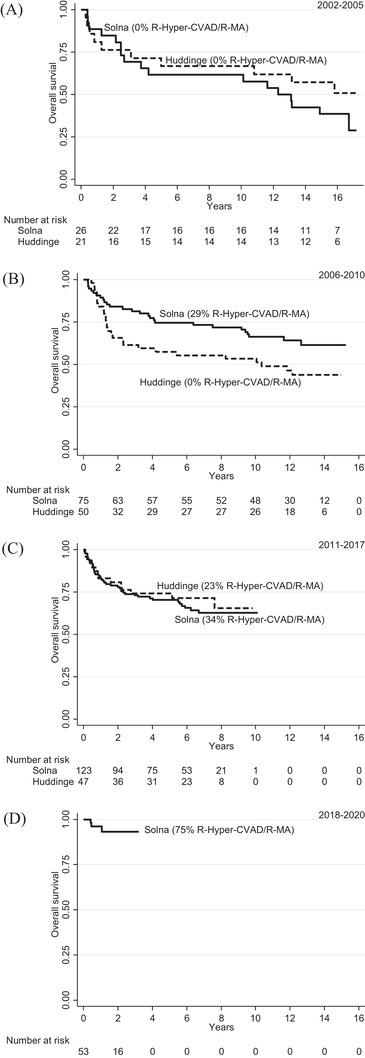
Overall survival in four calendar periods by academic site: (A) 2002–2005, (B) 2006–2010, (C) 2011–2017, (D) 2018–2020

To validate the superiority of R‐Hyper‐CVAD/R‐MA, we compared outcome between the two Karolinska sites, Solna and Huddinge, during the introduction of R‐Hyper‐CVAD/R‐MA. We allocated patients into four calendar periods, based on R‐Hyper‐CVAD/R‐MA use over time and sites: 2002–2005 (0% R‐Hyper‐CVAD/R‐MA use), 2006–2010 (29% R‐Hyper‐CVAD/R‐MA at Solna, 0% at Huddinge), 2011–2017 (34% R‐Hyper‐CVAD/R‐MA at Solna, 23% at Huddinge), 2018–2020 (75% R‐Hyper‐CVAD/R‐MA at Solna). OS was identical at the two sites 2002–2005, superior at Solna 2006–2010 (*p* = 0.044), identical again 2011–2017 and excellent at the Solna site 2018–2020 (*p* = 0.028 compared with previous periods; Figure 3). Survival was better 2018–2020, although there were more extreme high‐risk patients (aaIPI 3 53% vs. 37% in previous periods; *p* = 0.026). In 2018–2020, there were 53 patients who started therapy hospitalized at Solna, 44 with R‐Hyper‐CVAD and nine with R‐CHO(E)P), 11 of these have progressed (six after R‐Hyper‐CVAD [14%], five after R‐CHO(E)P [56%]) (PFS graphs in Figure S1). R‐Hyper‐CVAD/R‐MA patients became older over time, as shown above, and they also showed more WHO PS ≥2 (17%, 51%, 56%; *p *= 0.009) and overall clinical high‐risk characteristics over the three periods (aaIPI 3 9%, 35%, 51%; *p* = 0.0002; IPI 3–5 39%, 66%, 88%; *p *= 0.0004). However, outcomes in R‐Hyper‐CVAD patients were very similar in the three calendar periods (2‐year OS/PFS 88%/85%, 88%/81%, 98%/82%; Figure S2).

### 3.6 Outcome after lymphoma progression

There were 136 (34%) progressions after initiation of treatment, 65 of these progressed within 90 days of the final dose of first‐line therapy, and 41 of these were primary refractory. The 5‐year OS after progression was 25%; primary refractory lymphomas were significantly worse than others (5‐year OS 18% vs. 28%, *p* = 0.039). The median OS after progression was 8.4 months. First‐line regimen type did not influence outcome after progression. Eighteen of 261 patients starting with R‐CHO(E)P had CNS progression (7%) but only three of 129 starting with R‐Hyper‐CVAD (2%). CNS progression was predicted by CNS‐IPI 4–6 (*p* = 0.004), which was prognostic in both types of initial treatment. No patient with CNS progression survived 3 years. Of the 23 patients with CD5+ lymphoma 16 (70%) relapsed, of whom five (31%) showed CNS progressions. ASCT after salvage was conducted in 38 of 136 progressions (28%): in 21 of 69 progressions after R‐CHO(E)P (30%), eight of 17 (47%) after R‐CHO(E)P + consolidation, and seven of 18 (39%) after R‐Hyper‐CVAD/R‐MA, 0/3 (0%) after R‐ALL regimens and two of 29 (7%) after < 6 courses of immunochemotherapy. The 5‐year OS was 54% after ASCT. Another two patients underwent chimeric antigen receptor (CAR) T‐cell therapy as salvage.

## 4 DISCUSSION

This is, to our knowledge, the largest single‐centre series of R‐Hyper‐CVAD/R‐MA for aggressive B‐cell lymphoma. Our real‐world, retrospective study of all high‐risk hospitalized patients treated for aggressive B‐cell lymphoma at Karolinska between 2002 and 2020 shows that R‐Hyper‐CVAD/R‐MA is a very effective regimen. Although its superiority to R‐CHO(E)P was apparent in all clinical categories, it was most eye‐catching in patients with extreme high‐risk factors. We also saw that survival improved on group level at sites that started to use R‐Hyper‐CVAD/R‐MA, and it improved further when R‐Hyper‐CVAD/R‐MA became the dominant regimen.

An obvious limitation of our study is the inherent bias in comparing outcome after different regimens, since their selection was based on prognostic properties such as stage, CNS involvement, *MYC* translocation and age. For example, CNS involvement did not appear as a risk factor, because most patients who had it received R‐Hyper‐CVAD. However, outcome was much worse in the minority of CNS‐involved patients who started with R‐CHO(E)P (5‐year PFS 38% vs. 88%). CD5 positivity did not influence the choice of therapy and thus showed strong impact on outcome, which was disastrous after R‐CHO(E)P (5‐year PFS 7% vs. 66%). We saw a strong propensity for CD5+ DLBCL to progress, particularly in the CNS; this has also been shown by others. [13] We addressed selection bias by comparing outcomes between the two Karolinska sites and calendar periods with different R‐Hyper‐CVAD/R‐MA use, analysing all high‐risk patients on site/period level. Survival was better at the site that 2006–2010 was the only one using R‐Hyper‐CVAD/R‐MA (given to about a third of patients), was identical 2011–2017 when both sites used R‐Hyper‐CVAD/R‐MA (to a quarter or a third of patients) and improved greatly from 2018 when R‐Hyper‐CVAD/R‐MA became the dominant regimen (to three quarters of patients). Although clinical characteristics of R‐Hyper‐CVAD patients, such as age, PS and aaIPI, became increasingly adverse over time, outcomes among them were equally good in the three calendar periods (Figure S2); furthermore, this figure shows that also R‐CHO(E)P patients had very stable but inferior outcome, even during 2002–2005, when there could be no patient selection, because R‐CHO(E)P was the only existing treatment option.

Excepting CD5, this study has not addressed the underlying biological mechanisms that drive clinical behaviour in aggressive B‐cell lymphoma, and genetic investigations and cell‐of‐origin immunostainings were not conducted universally. [14, 15] Still, in all subgroups, there was a similar impression of better outcome with R‐Hyper‐CVAD (Figure 2), and 5‐year PFS was 70% in HGBCL (25% after R‐CHO(E)P [supplement]). Many biological properties appear to exert their effects partly through correlations with established clinical high‐risk markers such as disease stage, because it has been shown that patients with limited‐stage double‐hit lymphoma have good prognosis and maybe do not need intensive regimens such as R‐Hyper‐CVAD/R‐MA. [16] However, most double‐hit lymphoma‐patients do have widespread disease, and our results agree with others who have found that they relapse more seldom after intensive regimens. [17, 18, 19] There are emerging data beyond cell‐of‐origin that indicate a future of a more personalized approach in aggressive B‐cell lymphoma. [14] Trials which have added small molecules to R‐CHOP have not been successful, [20, 21, 22, 23] which might be partly due to the selection of less urgent cases into clinical trials (see this excellent review [24]). We think that R‐Hyper‐CVAD/R‐MA is a stronger immunochemotherapy backbone than R‐CHOP, at least for trials incorporating high‐risk patients with urgent need for intervention.

Our policy today is to start reasonably fit high‐risk aggressive B‐cell lymphoma patients < 70 years on R‐Hyper‐CVAD, and this study suggests that their expected 5‐year PFS is 77% (with IPI 4–5, 72%; with aaIPI 3, 69%). It should be emphasized that this large series of R‐Hyper‐CVAD/R‐MA‐treated patients is real‐world, including people with HIV, odd histologies, organ malfunctions and those who were diagnosed and started treatment at the intensive care unit. The median diagnosis‐to‐treatment interval was 8 days, a sign of urgency shown to correlate with adverse outcome. [10] This kind of patient is seldom included in prospective trials, because there is too little time to complete screening procedures. Nonetheless, we compared our data with three published prospective trials, the NLG CHIC, [25] the British R‐CODOX‐M/R‐IVAC [8] and MD Anderson's R‐Hyper‐CVAD/R‐MA trial [9] (comparisons in supplement). When we applied the inclusion criteria of each respective trial on our R‐Hyper‐CVAD cohort and investigated outcomes, we found very similar outcome in our patients compared with these three trials, although it seems that treatment‐related mortality in our older patients was lower, which we believe might be due to the flexibility outside trials to switch between regimens, and partly because our patients were treated at a single‐centre with long experience with the regimen. This probably also explains the differences with multi‐centre [26] and the similarities with single‐centre [27] studies of R‐Hyper‐CVAD/R‐MA. Still, R‐Hyper‐CVAD/R‐MA causes considerable toxicity, and there were six toxic deaths during induction therapy among the 150 patients who received at least one cycle of R‐Hyper‐CVAD/R‐MA, all in patients aged 54–65. Still, also older patients showed better outcome after R‐Hyper‐CVAD. Our results are in line with a growing body of evidence indicating the value of adding HD‐Mtx to patients with high‐risk aggressive B‐cell lymphoma. [28, 29] However, there are also reports which show little benefit from HD‐Mtx as a single agent, [30, 31] but from its part of intensive regimens such as R‐CODOX‐M/R‐IVAC and R‐Hyper‐CVAD/R‐MA. [32] We believe that HD‐Mtx and HD‐cytarabine together might be very important (the R‐MA part of the regimen), likewise with the hyper‐fractionated cyclophosphamide. With respect to long‐term toxicity, there were fewer cases of myeloid malignancy and heart disease after R‐Hyper‐CVAD/R‐MA than after R‐CHO(E)P; there are other series which show rather high frequencies of secondary myeloid malignancy after R‐CHOEP. [25, 33] Patients who relapsed after R‐Hyper‐CVAD/R‐MA were not more difficult to salvage than others. The low cumulative doxorubicin dose of 150–200 mg/m^2^ after 6–8 R‐Hyper‐CVAD/R‐MA allows us to incorporate anthracyclines into salvage regimens.

The improving outcome in our patients encourages us to continue to explore R‐Hyper‐CVAD/R‐MA for high‐risk patients in a prospective clinical trial. This is a highly toxic regimen, during which febrile neutropaenias are expected and not a cause for discontinuation. Some patients have more severe toxicity, and for these we might consider a reduction of intensity to R‐CHOP or R‐CHOEP. Between cycles, at home, every patient is since 2017 treated by an extensive network of advanced ambulatory hospital care‐givers, who may quickly see the patient, consult with the haematologist‐on‐call and initiate IV antibiotics and nutrition at home—many patients with febrile neutropaenias never need to be hospitalized. We believe that this helps explain our falling rates of life‐threatening complications, although R‐Hyper‐CVAD/R‐MA patients are older now. We have not seen a toxic death since 2017. We stress this because the safety of R‐Hyper‐CVAD/R‐MA is increased with improved control, not only at the hospital with respect to hydration, methotrexate concentrations et cetera, but also of patients’ wellbeing at home.

We conclude that R‐Hyper‐CVAD/R‐MA is a regimen that shows excellent OS and PFS in hospitalized high‐risk patients < 70 years with diffuse large B‐cell and other aggressive B‐cell lymphomas. The regimen has significant complications that should be managed with a rigorous monitoring system that maximizes safety during the entire period of induction. The emergence of novel treatments such as CAR T‐cells and bispecific antibodies gives relapsed/refractory patients with aggressive B‐cell lymphoma new hope. [24, 34] However, it is medically (and economically) wiser to give primary treatment that minimizes the relapse rates.

## CONFLICT OF INTEREST

The authors declare that they have no competing interests.

## AUTHOR CONTRIBUTIONS

Kristina Sonnevi, Maria Ljungqvist and Björn Engelbrekt Wahlin planned the project and gathered data. Per Bernell initiated the praxis of treating non‐Burkitt aggressive B‐cell lymphoma patients with R‐Hyper‐CVAD. Kristina Sonnevi and Björn Engelbrekt Wahlin wrote the manuscript. All authors designed the study and discussed its main conceptual idea, its results and contributed to the final version of the manuscript.

## Supporting information



Supporting Information
