# Supplementary material for: Excellent survival after R‐Hyper‐CVAD in hospitalized patients with high‐risk large B‐cell lymphoma: The Karolinska experience
Source: EJHaem. 2021 Sep 28;2(4):774–84. doi: 10.1002/jha2.296 (PMC9175941; doi:10.1002/jha2.296)
Supplement: Supplementary file 1 — Supporting Information [file JHA2-2-774-s001.pdf]

Supplement to Excellent survival after R-Hyper-CVAD in hospitalized patients with high-risk large B-cell lymphoma: the Karolinska experience

Supplementary methods

Immunochemotherapy regimens

R-CHOP and R-CHOEP are dosed IV rituximab 375 mg/m^2 day 1, IV cyclophosphamide 750 mg/m^2 day 1, IV doxorubicin 50 mg/m^2 day 1, IV vincristine 1.4 mg/m^2 (max 2 mg) day 1, PO prednisone 50 mg/m^2 day 1-5, adding to R-CHOEP IV etoposide 100 mg/m^2 day 1 and PO etoposide 200 mg/m^2 day 2-3. GCSF is given days 6-11. Cycles are repeated every 2 or 3 weeks. Blood samples weekly.

HD-cytarabine according to the first NLG trial[1] is dosed 3000 mg/m^2 (if <60 years) or 2000 mg/m^2 (if ≥60 years) 1 hr x 2 day 1-2.

HD-Mtx according to the first NLG trial[1] is dosed 3000 mg/m^2 (if <60 years) or 2000 mg/m^2 (if ≥60 years) 24 hrs day 1, calciumfolinate starts at 36 hrs (day 3).

R-Hyper-CVAD (course 1, 3, 5, 7 of R-Hyper-CVAD/R-MA) is dosed IV rituximab 375 mg/m^2 day 1, IV cyclophosphamide 300 mg/m^2 day 1, IV cyclophosphamide 300 mg/m^2 x 2 day 2-3, IV cyclophosphamide 300 mg/m^2 day 4, IV mesna 60 mg/m^2 prior to, and PO mesna 400 mg 2h and 6h after, each cyclophosphamide dose day 1-4. IT methotrexate 15 mg day 2, IV vincristine 2 mg day 4 and 11, IV doxorubicin 50 mg/m^2 day 4, PO dexamethasone 15

mg/m^2 day 1-4 and 11-14. The course is started when leukocytes >2/nL and platelets >50/nL. GCSF is given from day 7 until leukocytes >2/nL. Blood samples three times/week.

R-MA (course 2, 4, 6, 8 of R-Hyper-CVAD/R-MA) is dosed IV rituximab 375 mg/m^2 day 1, IV methotrexate 200 mg/m^2 2 hrs then IV methotrexate 800 mg/m^2 22 hrs day 1, calciumfolinate starts at 42 hrs (day 3), IT methotrexate 15 mg day 2, IV cytarabine 3000 mg/m^2 3 hrs day 2, IV cytarabine 3000 mg/m^2 3 hrs x 2 day 3, IV cytarabine 3000 mg/m^2 3 hrs day 4, PO dexamethasone 15 mg/m^2 day 1-3. The course is started when leukocytes >2/nL and platelets >50/nL and estimated glomerular filtration rate >50 mL/min. GCSF is given from day 7 until leukocytes >2/nL. Blood samples three times/week. Trimetoprim-sulpha is paused during methotrexate infusion.

Patients with leptomeningeal CNS involvement receive IT triplets twice weekly until liquor is free from lymphoma: IT 12 mg methotrexate, IT 30 mg cytarabine, IT 20 mg prednisolone.

Antimicrobial prophylaxis

Prophylactic treatment for *Pneumocystis* pneumonia was given to all patients throughout the study period. The standard treatment was trimethoprim/sulfamethoxazole 400 mg/80 mg qd. In case of intolerance, pentamidine inhalations or dapsone were offered. Antiviral prophylaxis was given against herpes simplex virus and varicella zoster virus to all patients: valaciclovir 250 mg bid as a continuous treatment. In R-Hyper-CVAD/R-MA, fungal prophylaxis was given during the neutropenic phase, from chemotherapy course day 7 and until ANC recovery. After R-Hyper-CVAD fluconazole 100 mg qd was given and after R-MA,

posaconazole 300 mg qd. Other antibiotic prophylaxis (e.g. gram-negative bacteria prophylaxis) was not given routinely but might be used in selected high-risk cases.

Supplementary results

Diffuse large B-cell lymphoma

The 285 patients with diffuse large B-cell lymphoma showed 5-year OS/PFS 66%/58%, and with first-course R-Hyper-CVAD 86%/78%, with R-CHO(E)P 62%/53%. In patients treated with full regimens, 5-year overall survival/progression-free survival (OS/PFS) was after R-Hyper-CVAD/R-MA 90%/78%, R-CHO(E)P + consolidation 73%/66%, and R-CHO(E)P 66%/58%. Five-year PFS in patients with germinal center and non-germinal center phenotypes were after R-Hyper-CVAD 82% and 72%, after R-CHO(E)P 54% and 46%.

Primary mediastinal B-cell lymphoma

Outcome in the 40 young patients (median age 35 [range, 18-68] years) with primary mediastinal B-cell lymphoma was excellent: 5-year PFS was 79% and R-Hyper-CVAD was the most common starting regimen (n = 20). R-Hyper-CVAD starters showed 5-year OS/PFS 95%/89%, R-CHO(E)P starters 89%/68%. In completed regimens, 5-year OS/PFS was after R-Hyper-CVAD/R-MA 100%/93% and R-CHO(E)P 85%/64% (consolidation was only given to one patient).

Other lymphoma subtypes

Of the other subtypes the most common was high-grade B-cell lymphoma, with 35 patients, which probably is an underestimation because assessments of *MYC*, *BLC2*, and *BCL6* translocations were not done in all patients. High-grade B-cell lymphoma was not a predictor of outcome compared with diffuse large B-cell lymphoma (5-year PFS 57% v. 58%; P = 0.25), likely because R-Hyper-CVAD was overrepresented among these 35 patients (20/35 started with R-Hyper-CVAD and 21 of 27 who completed first-line received R-Hyper-CVAD/R-MA). The 5-year PFS was, after completed regimens of R-Hyper-CVAD/R-MA and R-CHOEP, 70% and 25%, respectively. The other subtypes were too few for a meaningful analysis, but together they did not show different outcome than diffuse large B-cell lymphoma (P [PFS] = 0.27).

A comparison with the CHIC trial

Recently, the Nordic CHIC trial for primary treatment of high-risk aggressive lymphoma was published.[2] Major inclusion criteria were aggressive CD20+ B-cell lymphoma (not Burkitt or transformation), age <65 years, age-adjusted (aa) international prognostic index (IPI) 2-3 or involvement of testes/sinus/bone marrow or 2 or more extranodal sites, no cardiac/kidney/liver failure, no HIV positivity, WHO performance status (PS) <4. In the CHIC trial the median age was 56, and of the 139 patients 91% had lactate dehydrogenase (LDH) increased, 92% stage III-IV, 93% aaIPI 2-3. They were enrolled for treatment with 2 courses of R-CHOP+HD-Mtx, 4 R-CHOEP, 1 R-HD-cytarabine, resulting in 5-year OS/PFS 83%/81% and 2.2% CNS events; 23/139 (16%) experienced disease progression; there were 4 cases of secondary myeloid malignancy. In our cohort there were 91 patients starting with R-Hyper-CVAD who fulfilled the CHIC criteria; the median age of these 91 was 44, 91% had

elevated LDH, 93% stage III-IV, 93% aaIPI 2-3. Thus, excepting age, this subset was very similar to the patient population of the CHIC trial; furthermore, our subset also includes patients with all types of CNS involvement, while the CHIC trial only allowed for flow cytometry positive liquor, but excluded patients with clinical, radiologic, or cytologic signs of CNS involvement. CNS was involved at diagnosis in 17% of these R-Hyper-CVAD patients. The 91 patients starting with R-Hyper-CVAD showed 5-year OS/PFS 86%/80%. Disease progression occurred in 13 (14%) including 2 (2.2%) CNS events. There was one case of secondary myeloid malignancy.

A comparison with the UK NRCI CODOX-M/R-IVAC trial

The R-CODOX-M/R-IVAC regimen shares many similarities with R-Hyper-CVAD/R-MA, with dose-dense chemotherapy, CNS-penetrating and hyperfractionated alkylating agents. R-CODOX-M/R-IVAC has higher IV methotrexate doses but lower IV cytarabine doses and these two IV compounds are not given in the same course. Furthermore, it also incorporates etoposide and is limited to a total of 4 courses. The major criteria for the British R-CODOX-M/R-IVAC trial[3] were age <66 years, IPI 3-5, stage II-IV, no cardiac/kidney/liver failure, and diffuse large B-cell lymphoma or any morphological variant. We removed from our cohort follicular lymphoma grade 3B, lymphomatoid granulomatosis grade 3, gray-zone lymphoma, and post-transplant lymphoproliferative disorder, resulting in 75 of our patients who met these criteria and started with R-Hyper-CVAD. In the trial, 110 patients started treatment with R-CODOX-M/R-IVAC; median age was 50 years, 40% IPI 4-5, 54% WHO PS ≥2 and 9% CNS involvement. The trial showed 68% PFS at 2 years (67% at 4 years); their PFS was not inferior in patients with CNS involvement (2-year PFS 70%). Patients >50 years and WHO PS ≥2 had problems tolerating trial treatment and they showed 2-year PFS 44%. In our comparable 75 patients starting with R-Hyper-CVAD, the figures were similar with median

age 54, 47% IPI 4-5, 56% WHO PS ≥2, 16% CNS involvement. The 2- and 4-year PFS was 77% and 73% and in the 12 patients with CNS involvement 92% and 92%, while patients >50 years and WHO PS ≥2 (n = 23) showed 2- and 4-year PFS 78% and 72%; these numbers were probably higher than in the trial because 21/23 older patients received ≥6 courses of chemotherapy (14 of these received 6-8 cycles of R-Hyper-CVAD/R-MA and 3 received 2-5 cycles of R-Hyper-CVAD/R-MA + additional R-CHO(E)P; 4 received only a first R-Hyper-CVAD course and then continued with R-CHO(E)P). The 3-year PFS was identical in our 32 patients ≤50 years and in the trial: 75% in both populations (Amy Kirkwood, personal communication).

A comparison with MD Anderson's R-Hyper-CVAD trial

MD Anderson's randomized R-CHOP v. R-Hyper-CVAD/R-MA trial included patients with stage III-IV DLBCL and aaIPI ≥2 and age <61 years, excluding patients with CNS involvement, WHO PS ≥3, inadequate organ function, or uncontrolled infections.[4] They started 49 patients on R-Hyper-CVAD. Median age was 49, 45% had WHO PS 2, 94% elevated LDH, 37% aaIPI 3, 73% stage IV. In these patients, the 3-year PFS rate was 76%, but with a large age discrepancy: patients ≤45 had 3-year PFS 87% but those 46-60 70%, and high early treatment mortality (12%) in the patients 46-60. Fifty-two R-Hyper-CVAD patients in our cohort fulfilled the criteria for that trial. The median age was 44 years, 25% had WHO PS 2, 96% elevated LDH, 21% aaIPI 3, 90% stage IV. The 3-year PFS with R-Hyper-CVAD as the first cvcle of immunochemotherapy was 77%, in the 30 patients ≤45 years 85%, in the 22 patients 46-60 65%. There were 2 early deaths because of R-Hyper-CVAD/R-MA, both occurred in patients 46-60 (9%).

Supplementary Figure 1. Progression-free survival in four calendar periods by academic site (A) 2002-2005, (B) 2006-2010, (C) 2011-2017, (D) 2018-2020.

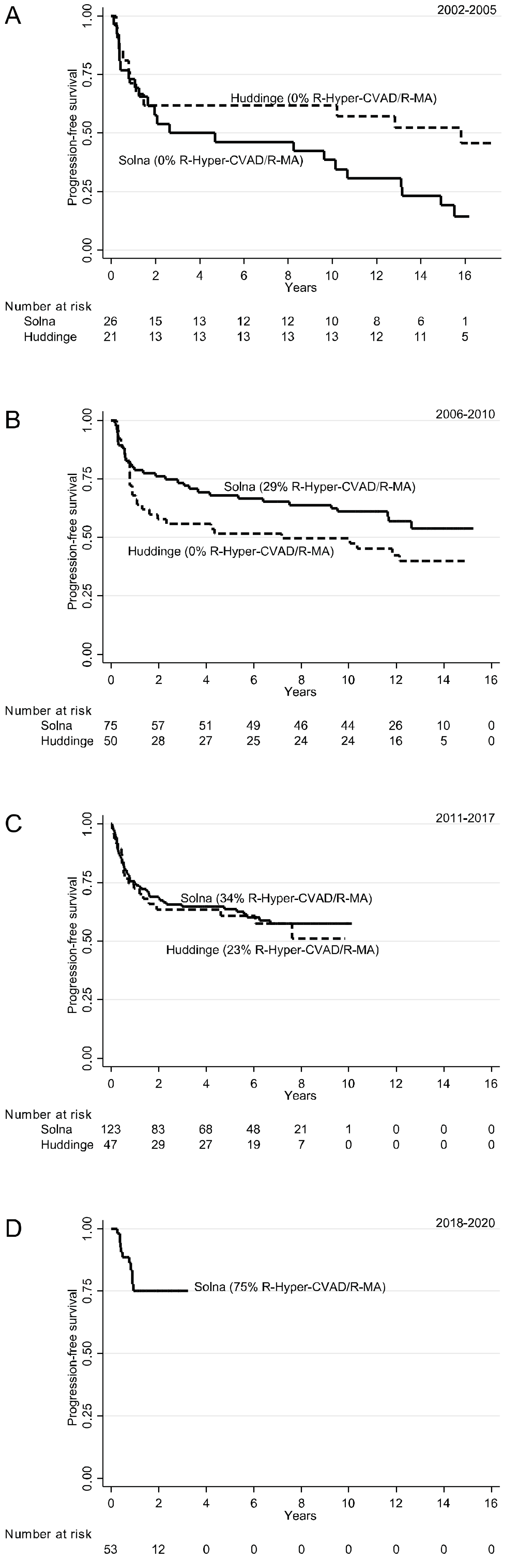

Supplementary Figure 2. Progression-free survival by calendar period and initial immunochemotherapy.

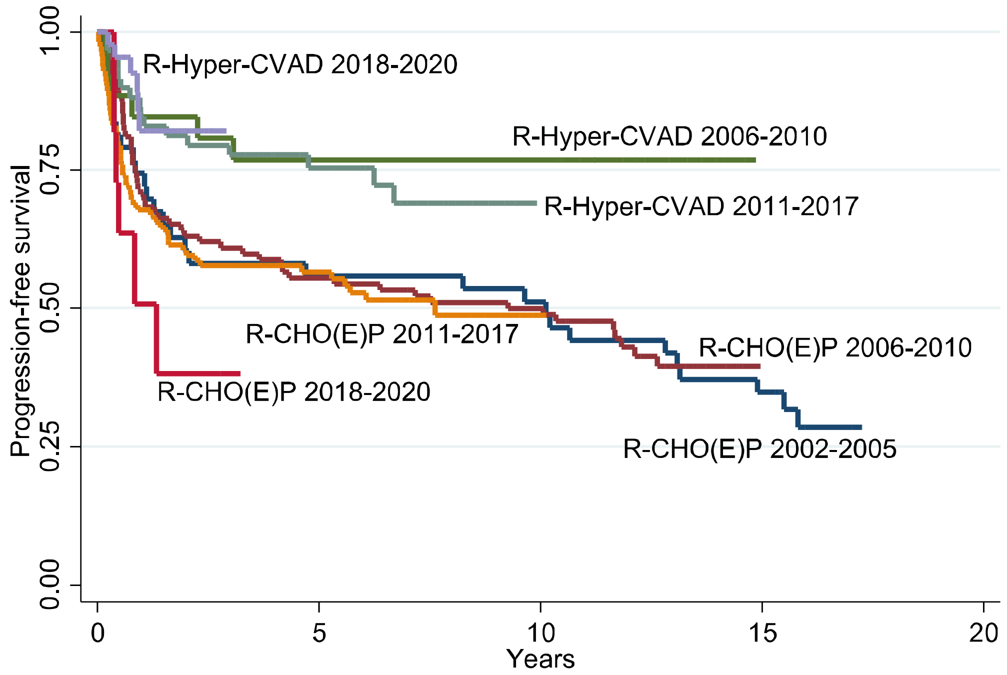

	0	5	10	15
R-CHO(E)P ’02-’05	43	24	22	15
R-CHO(E)P ’06-’10	95	51	45	0
R-CHO(E)P ’11-’17	112	48	1	0
R-CHO(E)P ’18-’20	11	0	0	0
R-Hyper-CVAD ’06-’10	26	20	20	0
R-Hyper-CVAD ’11-’17	59	31	0	0
R-Hyper-CVAD ’18-’20	44	0	0	0

References

1. Holte H, Leppa S, Bjorkholm M, et al. Dose-densified chemoimmunotherapy followed by systemic central nervous system prophylaxis for younger high-risk diffuse large B-cell/follicular grade 3 lymphoma patients: results of a phase II Nordic Lymphoma Group study. Ann Oncol 2013;24:1385-1392.
2. Leppa S, Jorgensen J, Tierens A, et al. Patients with high-risk DLBCL benefit from dose-dense immunochemotherapy combined with early systemic CNS prophylaxis. Blood Adv 2020;4:1906-1915.
3. McMillan AK, Phillips EH, Kirkwood AA, et al. Favourable outcomes for high-risk diffuse large B-cell lymphoma (IPI 3-5) treated with front-line R-CODOX-M/R-IVAC chemotherapy: results of a phase 2 UK NCRI trial. Ann Oncol 2020;31:1251-1259.
4. Oki Y, Westin JR, Vega F, et al. Prospective phase II study of rituximab with alternating cycles of hyper-CVAD and high-dose methotrexate with cytarabine for young patients with high-risk diffuse large B-cell lymphoma. Br J Haematol 2013;163:611-620.
